# Cigarettes' use and capabilities-opportunities-motivation-for-behavior model: a multi-country survey of adolescents and young adults

**DOI:** 10.3389/fpubh.2022.875801

**Published:** 2022-07-22

**Authors:** Heba Jafar Sabbagh, Wafaa Abdelaziz, Maryam Quritum, Nada AbuBakr AlKhateeb, Joud Abourdan, Nafeesa Qureshi, Shabnum Qureshi, Ahmed H. N. Hamoud, Nada Mahmoud, Ruba Odeh, Nuraldeen Maher Al-Khanati, Rawiah Jaber, Abdulrahman Loaie Balkhoyor, Mohammed Shabi, Morenike Oluwatoyin Folayan, Omolola Alade, Noha Gomaa, Raqiya Alnahdi, Nawal A. Mahmoud, Hanane El Wazziki, Manal Alnaas, Bahia Samodien, Rawa A. Mahmoud, Nour Abu Assab, Sherin Saad, Sondos G. Alhachim, Maha El Tantawi

**Affiliations:** ^1^Department of Pediatric Dentistry, Faculty of Dentistry, King Abdulaziz University, Jeddah, Saudi Arabia; ^2^Department of Pediatric Dentistry and Dental Public Health, Faculty of Dentistry, Alexandria University, Alexandria, Egypt; ^3^Faculty of Medicine, King Abdulaziz University, Jeddah, Saudi Arabia; ^4^Medical Faculty, Istanbul Medipol University, Istanbul, Turkey; ^5^City Quay Dental Practice and Implant Centre, Dundee, Scotland; ^6^Department of Education, University of Kashmir, Srinagar, India; ^7^Faculty of Dentistry, Alexandria University, Alexandria, Egypt; ^8^Faculty of Dentistry, National Ribat University, Khartoum, Sudan; ^9^College of Dentistry, Ajman University, Ajman, United Arab Emirates; ^10^Department of Oral and Maxillofacial Surgery, Faculty of Dentistry, Syrian Private University, Damascus, Syria; ^11^General Courses, King Abdulaziz University, Jeddah, Saudi Arabia; ^12^Faculty of Engineering, King Abdulaziz University, Jeddah, Saudi Arabia; ^13^King Saud bin Abdulaziz University for Health Sciences, Riyadh, Saudi Arabia; ^14^Department of Child Dental Health, Obafemi Awolowo University, Ile-Ife, Nigeria; ^15^Department of Preventive and Community Dentistry, Faculty of Dentistry, Obafemi Awolowo University, Ile-Ife, Nigeria; ^16^Schulich School of Medicine and Dentistry, University of Western Ontario, London, ON, Canada; ^17^Department of Dental Surgery, Oman Dental College, Muscat, Oman; ^18^UCSI University, Kuala Lumpur, Malaysia; ^19^Laboratory of Cereal Plant Pathology, National Institute for Argonomic Research, Settat, Morocco; ^20^Division of Imaging Science and Technology, School of Medicine, University of Dundee, Dundee, Scotland; ^21^Western Cape Education Department, Cape Town, South Africa; ^22^International Medical Centre, Jeddah, Saudi Arabia; ^23^Schools of Awqaf, Jerusalem, Israel; ^24^Gothenburg University, Gothenburg, Sweden; ^25^Health Education Services, Lansing, MI, United States

**Keywords:** smoking, anxiety, MPOWER, adolescents, young adults

## Abstract

The use of cigarettes among adolescents and young adults (AYA) is an important issue. This study assessed the association between regular and electronic-cigarettes use among AYA and factors of the Capability-Motivation-Opportunity-for-Behavior-change (COM-B) model. A multi-country survey was conducted between August-2020 and January-2021, Data was collected using the Global-Youth-Tobacco-Survey and Generalized-Anxiety-Disorder-7-item-scale. Multi-level logistic-regression-models were used. Use of regular and electronic-cigarettes were dependent variables. The explanatory variables were capability-factors (COVID-19 status, general anxiety), motivation-factors (attitude score) and opportunity-factors (country-level affordability scores, tobacco promotion-bans, and smoke free-zones) controlling for age and sex. Responses of 6,989-participants from 25-countries were used. Those who reported that they were infected with COVID-19 had significantly higher odds of electronic-cigarettes use (AOR = 1.81, *P* = 0.02). Normal or mild levels of general anxiety and negative attitudes toward smoking were associated with significantly lower odds of using regular-cigarettes (AOR = 0.34, 0.52, and 0.75, *P* < 0.001) and electronic-cigarettes (AOR = 0.28, 0.45, and 0.78, *P* < 0.001). Higher affordability-score was associated with lower odds of using electronic-cigarettes (AOR = 0.90, *P* = 0.004). Country-level-smoking-control policies and regulations need to focus on reducing cigarette affordability. Capability, motivation and opportunity factors of the COM-B model were associated with using regular or electronic cigarettes.

## Introduction

International associations recommend monitoring, screening, and preventing tobacco smoking among children and adolescents (1). Chronic exposure to tobacco is associated with bacterial proliferation, reduced periodontal immunity, and head and neck cancer (2, 3). Electronic cigarettes also deliver nicotine similar to cigarette smoking (4) and were suggested to cause cancer by affecting cell proliferation, periodontal cell migration, cytotoxicity, oxidative stress, apoptosis, and DNA maturation (5, 6). In addition, smoking affects oral and pharyngeal tissues causing irritation, stimulation of microbial growth, reduced periodontal tissue immunity, enhanced alveolar bone resorption, and delayed healing (3, 7). However, research is needed to identify differences between the use of regular and electronic cigarettes regarding determinants and effects.

In order to understand cigarette use, causes and control, multiple complex behavioral interventions need to be addressed. The Capability, Opportunity and Motivation for Behavior change (COM-B) framework (8) offers a structured approach for planning evidence-based interventions that could explain behavioral changes associated with cigarette use and control (8–12). The COM-B framework suggests that people engage in a behavior if they have the Capability (physical and psychological), the Opportunity, when the surrounding environment is physically and socially convenient and the Motivation to adopt this behavior. Within this framework, we conceptualized that the capability factors that may be associated with smoking during the study period include anxiety (13–15) and stresses caused by the COVID-19 pandemic (16) which are assumed to increase the risk of cigarettes' use (17, 18). Also, health problems such as COVID-19 infection may reduce the ability to smoke. Smoking is assumed to decrease in communities where there are legislative controls such as reducing access to cigarettes which decrease the opportunities to engage in smoking (18). The global variation in success of global strategies to control tobacco (18) emphasize the need for global evidence to inform tobacco control programs. Multi-country studies can shed light on person- and country-level factors associated with health problems. At the same time, assessing individual factors such as attitudes toward smoking can guide the development of tobacco cessation counseling activities.

The aim of this study was to assess the capability, opportunity and motivation factors associated with using regular and electronic cigarettes among adolescents and young adults (AYA) in several countries. The hypothesis of the study was that use of cigarettes was associated with capability, opportunity or motivation.

## Materials and methods

### Design and ethical consideration

A multi-country, online survey was conducted between August 2020 to January 2021. Ethical approval was obtained from the Research Ethics Committee of the Faculty of Dentistry, King Abdulaziz University in Saudi Arabia with Proposal No. 90-08-20. The study was conducted in accordance with the Helsinki Declaration (19).

### Participants

Participants were included if they were adolescents or young adults aged 11–23 years, if they/their parents consented to participate, if they were able to read the language of the survey, and if they had access to an electronic device with an Internet connection to access the survey. Exclusion criteria were children of younger age (below 11 years old), older adults (above 23 years-old), and children not living in the participating countries during the study period.

Sample size for estimating the prevalence of using regular or electronic cigarettes was based on the global prevalence of tobacco smoking for AYA. The prevalence ranged from 4.8 to 36.6% (20). The estimated sample size ranged from 71 to 356 participants. To calculate the sample size required to assess the factors associated with smoking, we used an online calculator for multilevel logistic regression models showing that with level 1 units (participants per country) at least = 50 and level 2 units (countries) = 25, the study would be able to detect at least moderate effect size (Cohen's d = 0.5) with power of 0.90 (21). Convenience sampling was used like in previous studies based on online surveys (22). Through snowball sampling, study participants were recruited and asked to distribute the survey link to others in their networks.

### Patient and public involvement

During the formation of the study survey tool, public and community members were contacted to seek their feedback. Also, a group of AYA who were not excluded from further analysis were interviewed for their opinion about the questionnaire. They were also involved in disseminating the survey to ensure reaching different level of the community.

### Study instrument development and validation

A questionnaire was developed with three sections. The first section assessed the socio-demographic information of the participant (age at last birthday, sex, and educational level) and parents' educational level.

The second section assessed participants' history of COVID-19 infection. Each participant was asked to identify if they had a history of diagnosed COVID-19 by checking a yes, no or do-not-know response. Also, the section included questions that assessed the psychological status of respondents using the Generalized Anxiety Disorder 7-item scale (GAD-7). The scale describes the frequency of seven items assessing general anxiety in the last 2 weeks (during the pandemic) on a 4-point scale ranging from 0: not at all to 3: nearly every day. The total score was the sum of points of all items ranging from 0 to 21. The cut-off points were 5, 10, and 15 for mild, moderate, and severe anxiety, respectively (23, 24). The scale had been validated for use in many of the countries included in the study (25, 26). The section also included five items specially developed for the study assessing respondents' attitude toward smoking asking participants whether they agreed with the following statements: that smoking leads to addiction, that it should be banned in children younger than 18 years old, that smokers are at greater risk of catching COVID-19, that they are at greater risk of having more severe COVID-19 manifestations and that it has harmful effects on the body. These items had three-point responses (agree, not sure, and disagree). The attitude score was created by recoding responses into agree (code 1) and others (code 0) and adding the points. Higher scores indicated more negative attitude toward smoking.

The third section enquired about regular and electronic cigarette use during the pandemic using the Global Youth Tobacco Survey (23). It asked about the current use of regular or electronic cigarettes (yes/no), the frequency of use (daily, weekly, monthly or less than monthly), changes in the frequency of use during the pandemic (less, same, or more than before) and the increase in nighttime use.

The questionnaire was developed in Arabic and translated into English, Turkish, Malay, and French by native researchers who were invited to collaborate with the core study team. The content validity index (CVI) for each version was calculated (27). The CVI for the Arabic and English versions was 0.87 based on evaluation by nine dentists, 0.97 for the Turkish version based on evaluation by seven dentists, 0.80 for the Malay version based on evaluation by five dentists and 0.88 for the French version based on evaluation by five dentists. Each version was pilot tested on 10 participants to ensure clarity and appropriate use of terms. The questionnaire was uploaded to the online platform SurveyMonkey® and the settings were managed to ensure that responses were anonymous and that responses could be submitted only once from the same electronic device. Cronbach alpha was used to assess the internal consistency of the GAD-7 and the attitude items. The Cronbach alpha of GAD-7 = 0.90 and that of the attitude to smoking scale = 0.63.

### Study procedure

At the beginning of the survey, participants were required to identify if they belonged to the age groups: 11–14, 15–18, or 19–23 year old (1, 28). Participants who were 11–14 or 15–18-year-old were directed to a form recording the parent's consent for their participation. At the end of the form, the consenting parent was asked to invite their child to respond to the survey without supervision and to assure the child of the confidentiality of their responses. Participants who were 19–23-year-old were able to consent before proceeding to the survey. The purpose of the study was explained to the participants, and they were assured that they were free to withdraw from the survey at any time.

The core team (King Abdulaziz University, Saudi Arabia, and Alexandria University, Egypt) invited collaborators in their own network to collect data in different countries. Repeated invitations were sent to ensure maximum coverage since data could only be collected by country-collaborators. Interested collaborators received the study protocol, ethical approval, and timeline for the study. A customized link and a responses-tracking link were created for each collaborator in the language of interest. Recruitment was also done through social media.

### Statistical analysis

There were two dependent variables for the study: the use of regular cigarettes and the use of electronic cigarettes. We used multi-level logistic regression analysis to account for the clustering of participants within countries. Level 1 factors were related to participants: participant's age and sex (confounders), COVID-19 infection status, GAD-7 score (capability factors) and attitude score (motivation factor). Level 2 factors represented opportunity factors measured at country level. Data for these factors were publicly available and obtained from the latest statistics included in the World Health Organization report on the tobacco pandemic in 2021 (29). These factors included the affordability score, which is the percentage of per capita gross domestic product required to buy 2,000 cigarettes of the most popular brand (29) with higher values indicating less affordable tobacco products. There was also a score ranging from 0 to 2 assessing the bans on tobacco promotion on television (29) including tobacco brands and tobacco products with higher score indicating greater compliance with bans. The last opportunity factor included a smoke-free zones score (29) assigning a point when smoking was banned in educational facilities except universities, in universities, restaurants and public transportation with a score ranging from zero to 4 with greater scores indicating greater availability of smoke-free areas. Level 1 and level 2 factors were fixed effect variables, and country of residence was entered as a random effect variable. We used robust estimation to address violations of model assumptions, fitted a random intercept and selected variance component as random effect covariance type. Adjusted odds ratios (AORs), 95% confidence intervals (CIs), and *P* values were calculated. We also used −2 log likelihood (−2LL) as a measure of deviance to assess the model goodness of fit with smaller values indicating better fit. We reported the −2LL for the unconditional model (without fixed effect variables) and for the conditional model where the fixed effect variables were included and calculated the difference between these two values then tested the significance of the difference using chi square test. We fitted unadjusted models where individual variables were introduced followed by adjusted models were all variables were introduced and mutually adjusted for each other. IBM SPSS for Windows version 22.0 (IBM Corp., Armonk, N.Y., USA) was used for analysis. Significance level was set at 5%.

## Results

Complete data were available for 6,989 participants from 25 countries (Supplementary Table 1). Table 1 shows that most participants were 18–23 years old (74.4%), females (57.3%), with a university/college degree (56.8%). Most participants' parents had university degrees (42.6% of mothers and 53.3% of fathers). Also, 529 (7.6%) participants were infected with COVID-19, 712 (10.2%) currently used regular cigarettes and 395 (5.7%) currently used electronic cigarettes.

**Table 1:** Sociodemographic, capability, opportunity and motivation factors (*n* = 6,989).

**Variables**		***N*** **(%)**
Age	18–23	5,200 (74.4)
	15–17	1,093 (15.6)
	11–14	696 (10.0)
Sex	Male	2,982 (42.7)
	Female	4,007 (57.3)
Education of participant	Elementary or less	236 (3.4)
	Middle school	582 (8.3)
	High school	2,202 (31.5)
	University/college degree	3,969 (56.8)
Mother's education	No education	560 (8.0)
	Finished elementary or middle school	1,494 (21.4)
	Finished high school	1,955 (28.0)
	Finished university studies or more	2,980 (42.6)
Father's education	No education	313 (4.5)
	Finished elementary or middle school	1,217 (17.4)
	Finished high school	1,737 (24.9)
	Finished university studies or more	3,722 (53.3)
Currently uses regular cigarettes	Yes	712 (10.2)
	No	6,277 (89.8)
Currently uses electronic cigarettes	Yes	395 (5.7)
	No	6,594 (94.3)
**Capability factors**		
Infected with COVID-19	Yes	529 (7.6)
	No	6,460 (92.4)
General anxiety levels	Normal	2,964 (42.4)
	Mild	2,621 (37.5)
	Moderate	900 (12.9)
	Severe	504 (7.2)
**Motivation factor**		
**Attitude score (out of 5): median (25th, 75th quartile)**		3 (2,4)
**Opportunity factors**		
Affordability score (%): median (25th, 75th quartile)		3.8 (3.0, 5.9)
Scoring of banning promotion on TV (out of 2): median (25th, 75th quartile)		2 (1,2)
Smoke free zones score (4): median (25th, 75th quartile)		3 (3,4)

Also, 2,964 (42.4%) of the participants had normal anxiety and 504 (7.2%) had severe anxiety. The median scores for attitude toward smoking scale = 3 out of 5, for banning promotion on television = 2 out of 2, and for smoke free zones = 3 out of 4. The median affordability score was 3.8%.

Table 2 shows that daily use was reported by 588 (82.6%) of regular cigarettes users and 213 (53.9%) electronic cigarettes users. Also, 363 (51.0%) and 174 (44.1%) participants reported the same frequency of using regular and electronic cigarettes as before the pandemic, 201 (28.2%) and 123 (31.1%) reported less frequent use during the pandemic; and 300 (42.1%) and 177 (44.8%) participants reported increased use of regular and electronic cigarettes at night.

**Table 2:** Characteristics of using regular cigarettes (*n* = 712) and electronic cigarettes (*n* = 395).

**Variables**		**Regular cigarettes** ***N*** **(%)**	**Electronic cigarettes** ***N*** **(%)**
Frequency of use	Daily	588 (82.6)	213 (53.9)
	Weekly	75 (10.5)	81 (20.5)
	Monthly	25 (3.5)	48 (12.2)
	Less than monthly	24 (3.4)	53 (13.4)
Change in frequency of use during COVID-19	Less than before COVID-19	201 (28.2)	123 (31.1)
	Same as before COVID-19	363 (51.0)	174 (44.1)
	More than before COVID-19	148 (20.8)	98 (24.8)
Increase in use at night	Yes	300 (42.1)	177 (44.8)
	No	412 (57.9)	218 (55.2)

Table 3 shows the factors associated with the current use of regular and electronic cigarettes in 25 countries. Participants who were 18–23-years-old and 15–17-years-old had significantly higher odds of using regular (AOR = 5.70, *P* < 0.001 and AOR = 2.28, *P* =0.005) and electronic cigarettes (AOR = 2.88, *P* < 0.001 and AOR = 2.25, *P* =0.002) than respondents who were 11–14-years-old. Male respondents had significantly higher odds of using regular (AOR = 5.96, *P* < 0.001) and electronic (AOR = 2.31, *P* < 0.001) cigarettes than females. Those who got infected with COVID-19 reported significantly higher odds of using electronic cigarettes (AOR = 1.81, *P* = 0.02). Having normal or mild levels of general anxiety were associated with significantly lower odds of using regular (AOR = 0.34 and 0.52, *P* < 0.001) and electronic (AOR = 0.28 and 0.45, *P* < 0.001) cigarettes. A higher score of negative attitudes toward smoking was associated with significantly lower odds of regular and electronic cigarettes use (AOR = 0.75 and 0.78, *P* < 0.001). A higher affordability score was associated with significantly lower odds of using electronic cigarettes (AOR = 0.90, *P* = 0.004). The fixed effect variables explained 23.9% of the random variation among countries in using regular cigarettes and 32.1% in using electronic cigarettes.

**Table 3:** Factors associated with using cigarettes and electronic cigarettes in AYA in 25 countries (*N* = 6,989).

**Factors**		**Regular cigarettes**				**Electronic cigarettes**			
		**UOR** **(95% CI)**	***P*** **-value**	**AOR** **(95% CI)**	***P*** **-value**	**UOR** **(95% CI)**	***P*** **-value**	**AOR** **(95% CI)**	***P*** **-value**
Age	18–23	4.96 (3.00, 8.19)	<0.001	5.70 (3.58, 9.06)	<0.001	2.88 (1.71, 4.83)	<0.001	2.88 (1.92, 4.31)	<0.001
	15–17	1.99 (1.14, 3.46)	0.02	2.28 (1.29, 4.02)	0.005	2.14 (1.22, 3.76)	0.008	2.25 (1.36, 3.72)	0.002
	11–14	1.00	–	1.00	–	1.00	–	1.00	–
Sex	Male	5.32 (4.41, 6.41)	<0.001	5.96 (4.31, 8.24)	<0.001	2.19 (1.76, 2.73)	<0.001	2.31 (1.55, 3.46)	<0.001
	Female	1.00	–	1.00	–	1.00	–	1.00	–
COVID-19 positive	Yes	1.14 (0.87, 1.49)	0.33	1.01 (0.75, 1.35)	0.97	1.95 (1.42, 2.66)	<0.001	1.81 (1.12, 2.92)	0.02
	No	1.00	–	1.00	–	1.00	–	1.00	–
General anxiety categories	Normal	0.47 (0.36, 0.63)	<0.001	0.34 (0.26, 0.46)	<0.001	0.34 (0.24, 0.49)	<0.001	0.28 (0.19, 0.43)	<0.001
	Mild	0.60 (0.45, 0.78)	<0.001	0.52 (0.44, 0.62)	<0.001	0.49 (0.35, 0.69)	<0.001	0.45 (0.30, 0.67)	<0.001
	Moderate	0.93 (0.68, 1.26)	0.63	0.95 (0.69, 1.30)	0.73	0.74 (0.50, 1.08)	0.12	0.69 (0.40, 1.21)	0.19
	Severe	1.00	–	1.00	–	1.00	–	1.00	–
Attitude score		0.74 (0.69, 0.78)	<0.001	0.75 (0.70, 0.80)	<0.001	0.78 (0.72, 0.84)	<0.001	0.78 (0.71, 0.86)	<0.001
Affordability score		0.99 (0.92, 1.07)	0.85	0.96 (0.92, 1.01)	0.09	0.90 (0.81, 0.99)	0.04	0.90 (0.83, 0.97)	0.004
Score of banning promotion on TV		1.21 (0.68, 2.15)	0.51	1.02 (0.53, 1.97)	0.94	0.59 (0.29, 1.22)	0.16	0.56 (0.25, 1.23)	0.15
Smoke free zones score		1.12 (0.90, 1.40)	0.31	1.17 (0.92, 1.49)	0.20	1.02 (0.75 1.38)	0.91	1.21 (0.96, 1.51)	0.10

*UOR: unadjusted odds ratio, AOR: adjusted odds ratio, CI: confidence interval*.

*In the adjusted models, the independent variables were mutually adjusted for each other*.

Variance estimate of unconditional model for regular cigarettes = 0.67 and for electronic cigarettes = 1.217, full model variance estimate = 0.51 and 0.826, respectively. Regarding goodness of fit, the deviance decreased from 40818.4 in the unconditional to 37424.7 in the conditional model where using regular cigarettes was the dependent variable and from 44525.3 to 42732.0 where using electronic cigarettes was the dependent variable with significant differences in both cases indicating improvement in model fit after adding the fixed effect variables.

## Discussion

The study showed that most current users of regular and electronic cigarettes reported either the same or lower level of use as before the pandemic. Older participants and males had higher odds of using regular and electronic cigarettes. Those with normal or mild anxiety and with negative attitude toward smoking had lower odds of reporting smoking. Participants with a history of COVID-19 infection and those from countries where electronic cigarettes were more affordable had higher odds of using electronic cigarettes. The components of COM-B were, thus, associated with the use of regular and electronic cigarettes.

One of the strengths of this study is the evidence generated on regular and electronic cigarettes use among AYA in several countries. Previous studies on using regular and electronic cigarettes during the pandemic had minor focus on AYA (30–32) or were restricted to only one country (16, 33–36) making it difficult to compare countries with different tobacco control policies. The use of the COM-B model as a conceptual framework for the study allowed the identification of individual and country-level factors that may be associated with smoking.

Despite the unique perspective offered by the present study, there were some limitations. First, there may be a social desirability bias in reporting smoking with potential for underestimating the level of smoking regular and electronic cigarettes. Second, the survey did not distinguish between the use of nicotine-containing and non-nicotine containing electronic cigarettes and this needs to be addressed in future studies. Third, because of COVID-19, data were collected electronically. The convenience sample may, therefore, not be representative of the profile of AYA in the countries included in the study as participants' recruitment was skewed toward those with access to the internet. Fourth, the study is cross-sectional and cannot prove causality; it can only suggest associations. Fifth, the attitude score was based on five items and this may possibly explain the relatively low level of internal consistency indicated by the Cronbach alpha (37) which might have also been reduced by the presence of more than dimension in the items assessing general attitude toward smoking and attitude toward the association of smoking and COVID-19 infection. Future studies assessing attitudes toward smoking may need to include a greater number of items to increase consistency. Despite the study limitations, there were important highlights from the study.

First, we found that a history of COVID-19 infection was associated with higher odds of using electronic cigarettes but not using regular cigarettes. This agrees with Gaiha et al. (16) who reported a significant association between smoking electronic cigarettes and history of COVID-19 infection. A recent meta-analysis of patients hospitalized for COVID-19 found no significant association between COVID-19 infection and smoking (38). However, this disagrees with some population-based studies (39). One possible explanation may be that AYA with a history of COVD-19 infection may opt to use electronic than regular cigarettes with the understanding that it would have less effect on their health. There are few studies that highlighted the interaction between smoking and COVID-19 (17). However, the association between electronic cigarette smoking and COVID-19 infection especially in AYA needs to be explored further. Electronic cigarettes smoking is associated with elevated chemokine CXCL8, extracellular matrix proteins and markers of mitochondrial dysfunction which decreases cellular viability and integrity. It also interferes with cellular mechanisms resulting in increased oxidative stress, inflammation, infections and airway remodeling in the lungs of device users (3, 38, 40–43). What is not known is whether this damage is worse for electronic cigarette smokers than it is for regular cigarette smokers; or if the damage is worse in AYA who smoke electronic cigarettes than it is for older smokers. These knowledge gaps need to be explored further. The study finding suggests that AYA who smoke electronic cigarettes should be counseled on the risk of contracting infection when sharing vaping devices (44).

Second, participants with severe anxiety had higher odds of using regular and electronic cigarettes. This agrees with studies reporting higher frequency of smoking to cope with stresses caused by the COVID-19 pandemic and the associated precautionary measures (45–47). These studies, however, mainly focused on adults and elderlies. We highlight that AYA equally need attention for psychological stress management even if they are less likely to have COVID-19 related deaths. Support provided to AYA should focus on helping them cope and maximize their resilience against surrounding stressors.

Third, less affordability of electronic cigarettes was associated with lower odds of smoking. Affordability is relevant for AYA because they may be less financially independent than adults (48). The rapid increase in electronic cigarette smoking among adolescence was associated with its affordability and availability (49–51). Further taxation of cigarettes during stressful events may be suggested to help reduce the risk of AYA using cigarettes to cope with stresses. However, it is important to note that capability and motivation factors may have stronger association with using electronic cigarettes than opportunity factors based on the present findings.

Fourth, banning tobacco promotion on television seemed to have a strong but non-significant negative association with using electronic cigarettes in the present study. This may be attributed to the impact of role modeling (52) on AYA.

The findings have implication for controlling tobacco smoking in AYA. At the individual level, educating AYA to manage stress without resorting to smoking, and promoting a negative attitude toward smoking may help control smoking. At a country level, it is important to ensure greater financial obstacles so that AYA are less able to afford tobacco products and to reduce the promotion of tobacco through advertisements in addition to exploring other policies. Adopting the MPOWER policies can help control smoking during the pandemic. The MPOWER package includes monitoring tobacco use and prevention policies (M), protecting populations from tobacco (P), offering help to quit tobacco (O), warning about the risks associated with tobacco (W), enforcing bans on tobacco promotion (E) and raising taxes on tobacco products (R). The package was created by the World Health Organization to help governments address the tobacco pandemic based on the framework convention for tobacco control (18). Enforcing the components of MPOWER may help reshape the surrounding environment, thus reducing the opportunities to engage in smoking as a behavior. The evidence generated in the present study supports the adoption of the MPOWER strategies to control smoking associated with stressful events. Further studies are needed to assess the impact of other MPOWER strategies on smoking, and smoking prevalence in AYA compared to other age groups.

## Conclusion

The higher odds for AYA to smoke in association with anxiety highlight the need for action to reduce the capability, opportunity and motivation of AYA to smoke during stressful events. The adoption of the MPOWER strategies may provide the framework for governments to address the structural determinants of smoking thereby supporting global tobacco control efforts.

## Data availability statement

The raw data supporting the conclusions of this article will be made available by the authors, without undue reservation.

## Ethics statement

The studies involving human participants were reviewed and approved by Research Ethics Committee of the Faculty of Dentistry, King Abdulaziz University, Jeddah Saudi Arabia. Written informed consent to participate in this study was provided by the participants' legal guardian/next of kin.

## Author contributions

HS, WA, MQ, and ME: conceptualization. MQ and ME: formal analysis. HS, MQ, NAA, JA, NQ, SQ, AH, NM, RO, NA-K, RJ, AB, MS, MF, OA, NG, RA, NAM, HE, MA, BS, RM, NA, SS, SA, and ME: investigation. HS, MQ, and ME: methodology. HS, WA, and ME: project administration and writing—original draft. HS, WA, NA-K, MF, and ME: writing—review & editing. All authors contributed to the article and approved the submitted version.

## Conflict of interest

The authors declare that the research was conducted in the absence of any commercial or financial relationships that could be construed as a potential conflict of interest.

## Publisher's note

All claims expressed in this article are solely those of the authors and do not necessarily represent those of their affiliated organizations, or those of the publisher, the editors and the reviewers. Any product that may be evaluated in this article, or claim that may be made by its manufacturer, is not guaranteed or endorsed by the publisher.
