# Supplementary material for: Cigarettes' use and capabilities-opportunities-motivation-for-behavior model: a multi-country survey of adolescents and young adults
Source: Front Public Health. 2022 Jul 22;10:875801. doi: 10.3389/fpubh.2022.875801 (PMC9353685; doi:10.3389/fpubh.2022.875801)
Supplement: Supplementary file 1 [file Table_1.DOCX]

Supplementary Material

# Supplementary Table

| **Country** | **Participants: n (%)** |
| --- | --- |
| Canada | 206 (2.9) |
| Egypt | 576 (8.2) |
| India | 491 (7.0) |
| Iraq | 100 (1.4) |
| Jordan | 80 (1.1) |
| Kuwait | 576 (8.2) |
| Lebanon | 67 (1.0) |
| Libya | 108 (1.5) |
| Malaysia | 138 (2.0) |
| Morocco | 121 (1.7) |
| Nigeria | 288 (4.1) |
| Oman | 188 (2.7) |
| Pakistan | 148 (2.1) |
| Palestine | 89 (1.3) |
| Philippines | 215 (3.1) |
| Saudi Arabia | 919 (13.1) |
| South Africa | 101 (1.4) |
| Sudan | 502 (7.2) |
| Sweden | 67 (1.0) |
| Syria | 400 (5.7) |
| Turkey | 682 (9.8) |
| United Arab Emirates | 444 (6.4) |
| United Kingdom | 225 (3.2) |
| United States | 50 (0.7) |
| Yemen | 208 (3.0) |
